# Intramuscular Cyanocobalamin Treatment in Patients with Corpus Atrophic Gastritis and Vitamin B_12_ Deficiency: Efficacy and Predictors of Increased Requirement—A Monocentric Longitudinal Real-Life Cohort Study

**DOI:** 10.3390/nu18020271

**Published:** 2026-01-14

**Authors:** Francesco Paolo Schiavone, Giulia Pivetta, Silvia Scalamonti, Manuela Pompili, Micaela Magnante, Gianluca Esposito, Bruno Annibale, Edith Lahner

**Affiliations:** Digestive Disease Unit, Department of Medical-Surgical Sciences and Translational Medicine, Sant’Andrea Hospital, Sapienza University of Rome, Via Grottarossa 1035, 00189 Rome, Italy; francescopaolo.schiavone@uniroma1.it (F.P.S.); giulia_pivetta@libero.it (G.P.); silvia.scalamonti@uniroma1.it (S.S.); mpompili@ospedalesantandrea.it (M.P.); mimagnante@ospedalesantandrea.it (M.M.); gianluca.esposito@uniroma1.it (G.E.); bruno.annibale@uniroma1.it (B.A.)

**Keywords:** atrophic gastritis, cobalamin deficiency, cyanocobalamin treatment, pernicious anemia, vitamin B_12_ deficiency

## Abstract

**Background and Objectives**: Corpus atrophic gastritis (CAG) is associated with vitamin B_12_ deficiency due to impaired gastric acid and intrinsic factor secretion. Untreated vitamin B_12_ deficiency can lead to pernicious anemia, severe neurological consequences, and acute cardiocerebral-vascular events. Timely vitamin B_12_ supplementation is relevant; however, the dosage of intramuscular (IM) vitamin B_12_ supplementation has not been standardized to date. The objective was to assess the efficacy of a 1st and 2nd treatment schedule of IM-cyanocobalamin treatment in CAG patients with vitamin B_12_ deficiency at long-term follow-up and to identify the predictors of increased cyanocobalamin requirement. **Methods**: This monocentric real-life cohort study included 213 CAG patients with vitamin B_12_ deficiency. Inclusion criteria were adult age, histological diagnosis of CAG with vitamin B_12_ deficiency (<220 pg/mL), and follow-up of more than 12 months. The 1st-treatment-schedule (TxA) was 5000 µg IM cyanocobalamin every 5 days for 3 times, followed by 5000 µg IM cyanocobalamin every 3 mos (20,000 µg/yr); the 2nd-treatment-schedule (TxB) was 5000 µg IM cyanocobalamin every 5 days for 3 times, followed by 5000 µg IM cyanocobalamin every 2 mos (30,000 µg/yr). The treatment endpoint was serum vitamin B_12_ normalization. Clinical-biochemical follow-up was scheduled every 12 ± 6 mos: patients who satisfied the endpoint maintained the TxA, otherwise, TxB was prescribed. **Results**: Of the 213 CAG patients with vitamin B_12_ deficiency, 48.3% had anemia, and 26.3% macrocytosis without anemia. TxA efficaciously corrected vitamin B_12_ deficiency in 146 (68.5%) patients, maintaining efficacy until the longest available follow-up (42.2 ± 2.6 months). The remaining 67 patients (31.5%) were switched to TxB due to persistent vitamin B_12_ deficiency observed at 12 (6–36) months and were maintained until the longest available follow-up (50.2 ± 4.1 months). At the longest available follow-up, a significant increase in Hb (TxA: 11.9 ± 0.2 to 13.1 ± 0.1 g/dL, *p* < 0.001; TxB: 12.2 ± 0.3 to 13.6 ± 0.2 g/dL, *p* = 0.003) and serum vitamin B_12_ (TxA: 168 ± 7 to 402 ± 19 pg/mL, *p* < 0.0001; TxB: 157 ± 12 to 340 ± 24 pg/mL, *p* < 0.0001) was shown in both schedules. A significant decrease in MCV was shown in TxB only (*p* = 0.0003). In logistic regression, switching to TxB was significantly associated with severe corpus intestinal metaplasia (OR 11.0, 95% CI 2.8–43.7), macrocytosis at CAG diagnosis (OR 2.7, 95% CI 1.2–6.3), and male sex (OR 2.4, 95% CI 1.1–5.2). **Conclusions**: In this real-world setting, at long-term follow-up, nearly 70% of CAG patients with vitamin B_12_ deficiency restored their vitamin B_12_ levels with 20,000 µg/yr of cyanocobalamin, while the remaining 30% required 30,000 µg/yr. Male vitamin B_12_-deficient CAG patients with advanced gastric damage and severe macrocytosis required higher dosages of cyanocobalamin. They should be carefully monitored to avoid suboptimal supplementation and potentially dangerous consequences of vitamin B_12_ deficiency.

## 1. Introduction

Corpus atrophic gastritis (CAG) is a histopathological entity characterized by chronic inflammation of the gastric mucosa associated with the progressive reduction or complete loss of oxyntic glands due to gastric autoimmunity or longstanding *Helicobacter pylori* infection. The loss of these glands, which are responsible for acid secretion, leads to impaired gastric acid secretion, an increase in gastric pH, and a reduced synthesis of intrinsic factor. Gastric acid secretion and intrinsic factor are both essential for vitamin B_12_ absorption, and gastric acid secretion is required for iron absorption. Therefore, the absorption of vitamin B_12_ and/or iron becomes impaired, thereby promoting the development of vitamin B_12_ deficiency and iron deficiency. These conditions are often responsible for the main clinical manifestations of CAG. Corpus atrophy may be corpus-restricted, with a spared antrum displaying the typical histopathological features of autoimmune atrophic gastritis; however, it may also be associated with antral atrophy, as typically occurs when *Helicobacter pylori*-related [1]. CAG patients are at increased risk for gastric neoplasms, such as gastric cancer and type 1 gastric neuroendocrine tumors; therefore, regular endoscopic surveillance is warranted [1,2,3,4].

Vitamin B_12_ deficiency may lead to the development of megaloblastic anemia (known as pernicious anemia), elevated blood levels of homocysteine, and demyelination of peripheral nerves. The main symptoms include asthenia, paraesthesia, dyspepsia, infertility and neuropsychiatric symptoms such as irritability, cognitive impairment and psychomotor retardation. This clinical picture is frequently insidious in onset and largely nonspecific, leading to delay in diagnosis [5,6,7,8,9].

In CAG patients, the treatment of choice for vitamin B_12_ deficiency involves supplementing cobalamin in its molecular forms, such as cyano-, hydroxo-, or methylcobalamin, either orally or via intramuscular (IM) injection. This regimen typically starts with a loading dose followed by lifelong maintenance therapy [5,7]. A few studies comparing the two administration routes have not demonstrated substantial differences in efficacy. Although oral treatment is less expensive and easier for patients to use, IM therapy is generally preferred at diagnosis or during follow-up to more rapidly restore cobalamin levels, especially in patients with severe cobalamin deficiency and/or neurologic symptoms. In any case, the IM route should be preferred in patients with vitamin B_12_ deficiency due to CAG to overcome intrinsic factor deficiency, which impairs intestinal absorption [10,11]. However, to date, scientific evidence on this topic remains limited; in particular, data on the optimal dosage of cobalamin supplementation are lacking, and current treatment schedules are based on the clinical experience of experts in this field [12,13,14]. One proposed treatment schedule consists of an IM injection of 1000 μg cyanocobalamin or hydroxocobalamin, administered every other day for one to two weeks, followed by weekly injections for one month, and then gradually tapered to once a month for lifelong use [14].

In clinical practice, cobalamin supplementation is generally individualized according to the patient’s clinical profile, preferences, and local availability of molecular forms. Anyhow, taking into account that only 10%–15% of the injected dose is retained, higher cobalamin dosages might be useful [13,14], but data on this issue are lacking.

Based on this background, the current study aimed to assess the efficacy of a 1st and a 2nd treatment schedule of IM cyanocobalamin treatment in CAG patients with vitamin B_12_ deficiency at long-term follow-up, as well as predictors of increased cyanocobalamin requirement.

## 2. Methods

This paper was drafted in accordance with STROBE guidelines [15].

### 2.1. Study Design and Population

A monocentric real-life cohort study was conducted on 213 patients (63% female, median age 63, range 20–86, 95% CI for the median 60–65 years) diagnosed between 2007 and 2021 with CAG and concomitant vitamin B_12_ deficiency in a teaching hospital, a referral centre for gastric autoimmunity and micronutrient malabsorption. Inclusion criteria were adult age (>18 years), histological diagnosis of CAG according to the updated Sydney system [16] with serum vitamin B_12_ deficiency (<220 pg/mL) with or without macrocytosis (mean corpuscular volume, MCV > 100 fl), anemia (hemoglobin, Hb < 13 g/dL males, <12 g/dL females) [17], IM cyanocobalamin supplementation treatment and a minimum follow-up of twelve months. Exclusion criteria were previous gastric surgery, lack of vitamin B_12_ deficiency or histological confirmation of CAG at follow-up investigations, non-adherence to cyanocobalamin treatment or follow-up, or incomplete data. According to these criteria, the included cohort of 213 patients derived from the original cohort of 710 patients with consecutively diagnosed CAG by excluding 497 (70%) patients; reasons for exclusion were absence of vitamin B_12_ deficiency (*n* = 210), lack of follow-up (*n* = 159) or lack of adherence to cobalamin supplementation treatment (*n* = 4), lack of confirmation of CAG at follow-up (*n* = 17), and incomplete data (*n* = 107).

As recommended by current European guidelines [2,3], endoscopic-histological surveillance was proposed at three-year intervals, unless new symptoms or the onset of anemia and/or iron deficiency occurred, in which case endoscopy was immediately indicated.

A database was built, including demographics, clinical, laboratory, and histological data, as well as supplementation treatment with cyanocobalamin. In particular, clinical data were collected to assess other possible causes of cobalamin deficiency, such as a vegan diet, malnutrition, pancreatic diseases, small intestine surgery, chronic use of proton pump inhibitors, H_2_ blockers, metformin, or cholestyramine [7]. The presence of one of these confounding factors was not considered a criterion for exclusion.

### 2.2. Treatment Schedules for Vitamin B_12_ Supplementation

Vitamin B_12_ supplementation was prescribed by the IM route according to clinical practice in our teaching hospital: The 1st treatment schedule (TxA) consisted of 5000 µg IM cyanocobalamin every five days for three times, followed by 5000 µg IM every three months (20,000 µg yearly). The 2nd treatment schedule (TxB) consisted of 5000 µg IM cyanocobalamin every five days for three times, followed by 5000 µg IM cyanocobalamin every two months (30,000 µg yearly). The treatment endpoint was serum vitamin B_12_ normalization. The efficacy of the treatment schedule was evaluated at clinical-biochemical follow-up outpatient visits scheduled at 12 ± 6 months. Complete blood cell count, serum cobalamin, and homocysteine levels were assessed at baseline and follow-up visits. Patients who satisfied the endpoint (normalization of serum vitamin B_12_ levels) maintained the first treatment schedule (TxA); otherwise, when the first treatment schedule (TxA) was insufficient to restore normal serum vitamin B_12_ levels, the second treatment schedule (TxB) was prescribed.

Clinical, biochemical, and histological data at the longest available follow-up were compared to the start of treatment in patients on both schedules and between schedules to find out predictors for the need for treatment change to achieve satisfaction of endpoints. All patients provided informed consent, and the study was approved by the ethics committee of Sapienza University, Sant’Andrea Hospital (No. 7022/2020, approval date: 23 July 2020).

### 2.3. Diagnostic Criteria for Corpus Atrophic Gastritis, Vitamin B_12_ Deficiency, and Follow-Up

Gastroscopies were performed by fully trained gastroenterologists and endoscopists. All patients underwent pharyngeal anesthesia (xylocaine spray puffs) and conscious intravenous sedation (midazolam 3–5 mg). Gastroscopies were performed using white light mode and, when available, with virtual chromoendoscopy (narrow-band imaging). Biopsies were collected according to the updated Sydney system [16], consisting of two biopsies from the antrum, one from the *incisura angularis*, and two from the corpus, and were sent for histopathological assessment in separate vials. In case of endoscopically visible mucosal abnormalities, further biopsies were taken. CAG was defined by histopathology assessed on gastric biopsies according to the updated Sydney [16] and diagnosed when gastric mucosal atrophy with or without intestinal metaplasia was present in the corpus mucosa; antral mucosa could be diagnosed with atrophy or could be spared as occurs in autoimmune atrophic gastritis [6,9]. The eventual presence of *Helicobacter pylori* infection was also assessed by histopathology, and, when present, eradication treatment was prescribed. Antibodies against parietal cells were positive in 149 (69.9%) patients, and antibodies against intrinsic factor in 54 (25.3%) patients.

For the purpose of the current study, the presence of serum vitamin B_12_ deficiency was defined as less than 220 pg/mL, with or without the concomitant presence of macrocytosis (MCV > 100 fl) or anemia (Hb < 13 g/dL males, <12 g/dL females) [6,7,17]. Since anemia was not present in all cases with CAG and vitamin B_12_ deficiency (a condition also defined as “subclinical pernicious anemia”), the term “pernicious anemia” was not used to avoid confounding.

After the diagnosis of CAG, all patients were informed about the increased risk of gastric neoplasms (gastric adenocarcinoma and type 1 gastric neuroendocrine tumors) by an information sheet and invited to follow an endoscopic-histological surveillance programme at three-year intervals [2,3]. When new upper gastrointestinal symptoms or anemia occurred, immediate gastroscopic investigation was indicated. Treatment with proton pump inhibitors or H_2_ blockers at the time of CAG diagnosis was withdrawn.

### 2.4. Treatment Endpoint of Vitamin B_12_ Supplementation

The treatment schedules with IM cyanocobalamin were considered efficacious when serum cobalamin (>220 pg/mL) was normalized. Haemoglobin, MCV, and homocysteine levels were also monitored, but their normalization (MCV 80–100 fl, homocysteine < 13.5 μmol/L) was not considered necessary for achieving the treatment endpoint.

### 2.5. Statistical Analyses

Descriptive statistics were performed using mean ± SEM, median (range, 95% CI for the median) for quantitative variables. The frequencies and percentages were computed for qualitative variables. To compare quantitative variables between groups, Student’s *t*-test was used. To compare qualitative (dichotomous) variables between groups, Chi-square test was used. To assess potential predictors of the need for a treatment schedule change, a logistic regression analysis (enter and stepwise methods) was performed, considering the 2nd treatment schedule (TxB) as the dependent variable and independent variables that showed significant differences at univariate analyses or were potentially clinically relevant or potential confounders. Predictors (independent variables significantly associated with the dependent variable) were expressed as OR (95%CI). A *p*-value of less than 0.05 was considered statistically significant. Statistical analyses were performed with MedCalc Statistical Software 22.009 (MedCalc Software, Ostend, Belgium; http://www.medcalc.org; 1 January 2023).

## 3. Results

As shown in Table 1, of the 213 included patients with CAG and concomitant vitamin B_12_ deficiency, 63% were female, and the median age was 63 years (range 20–86, 95% CI for the median 60 to 65 years). Anemia was present in 48.3% and was severe in 7.8%, while macrocytosis without anemia was found in 26.3%. Mean serum cobalamin levels were 164 ± 6 pg/mL, 41.7% and 28.1% had serum cobalamin levels of less than 200 pg/mL or 100 pg/mL, respectively. Additionally, 73.6% patients had cobalamin supplementation treatment before diagnosis of CAG. Concomitant iron deficiency was present in 54.8%.

Concerning other potential causes of vitamin B_12_ deficiency, the mean body mass index ± SEM was 25.7 ± 0.3 kg/m^2^; thus, there were no malnourished patients amongst the study population, 11.4% of patients were on metformin treatment, and 6.7% experienced previous abdominal surgery (cholecystectomy, colectomy, gynecological or obstetric surgery), all without small intestine resections. None of the patients reported chronic alcohol abuse, vegan diet, pancreatic diseases, chronic use of proton pump inhibitors, H_2_ blockers, or cholestyramine.

Regarding the histopathological features of CAG, 73.4% had corpus-restricted atrophy with a spared antrum, thus displaying the typical features of autoimmune atrophic gastritis. Corpus atrophy was severe in 73.9% of patients, and corpus intestinal metaplasia was present in 86.8% of patients. *Helicobacter pylori* infection was identified at histology in 31.9% of cases and was successfully treated with eradication therapy in all cases. Overall, gastric neoplastic lesions were detected at baseline or follow-up in 10.8% of patients, which were histopathologically characterized as type 1 gastric neuroendocrine tumors in 6.1% and as gastric adenocarcinoma in 4.7% of cases. The longest mean ± SEM follow-up of the 213 included CAG patients with vitamin B_12_ deficiency was 44.7 ± 2.2 months. Table 1 summarizes the main clinical and histological data of the 213 CAG patients with vitamin B_12_ deficiency.

### Supplementation Treatment with Intramuscular (IM) Cyanocobalamin

Of the 213 patients with CAG and vitamin B_12_ deficiency, 146 (68.5%) maintained the 1st treatment schedule (TxA), normalizing serum cobalamin levels on this treatment until the longest available follow-up. In contrast, 67 (31.5%) required a switch to the 2nd treatment schedule (TxB) at a median follow-up of twelve (range 6–36) months to achieve normalization of normal serum cobalamin levels; this treatment schedule was then maintained until the longest available follow-up. The longest available follow-up was similar in both treatment groups: TxA mean ± SEM 42.2 ± 2.6 months, TxB mean ± SEM 50.2 ± 4.1 months, *p* = 0.092.

In the TxA group, mean ± SEM Hb levels significantly increased from 11.9 ± 0.2 to 13.1 ± 0.1 g/dL (*p* < 0.001), mean ± SEM MCV slightly decreased from 88.8 ± 1.2 to 86.0 ± 0.7 fl without reaching statistical significance (*p* = 0.069), mean ± SEM homocysteine levels significantly reduced from 16.8 ± 0.9 to 12.3 ± 0.5 μmol/L (*p* = 0.0001), and mean ± SEM cobalamin levels significantly increased from 168 ± 7 to 402 ± 19 pg/mL (*p* < 0.0001). In the TxB group, mean ± SEM Hb levels significantly increased from 12.2 ± 0.3 to 13.6 ± 0.2 g/dL (*p* = 0.003), mean ± SEM MCV significantly decreased from 96.6 ± 1.9 to 88.6 ± 0.9 fl (*p* = 0.0003), mean ± SEM homocysteine levels significantly reduced from 28.5 ± 3.9 to 13.8 ± 0.9 μmol/L (*p* = 0.0002), and mean ± SEM cobalamin levels significantly increased from 157 ± 12 to 340 ± 24 pg/mL (*p* < 0.0001).

**Figure 1:**
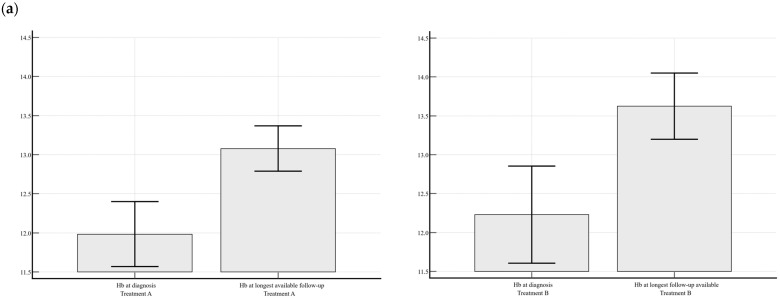

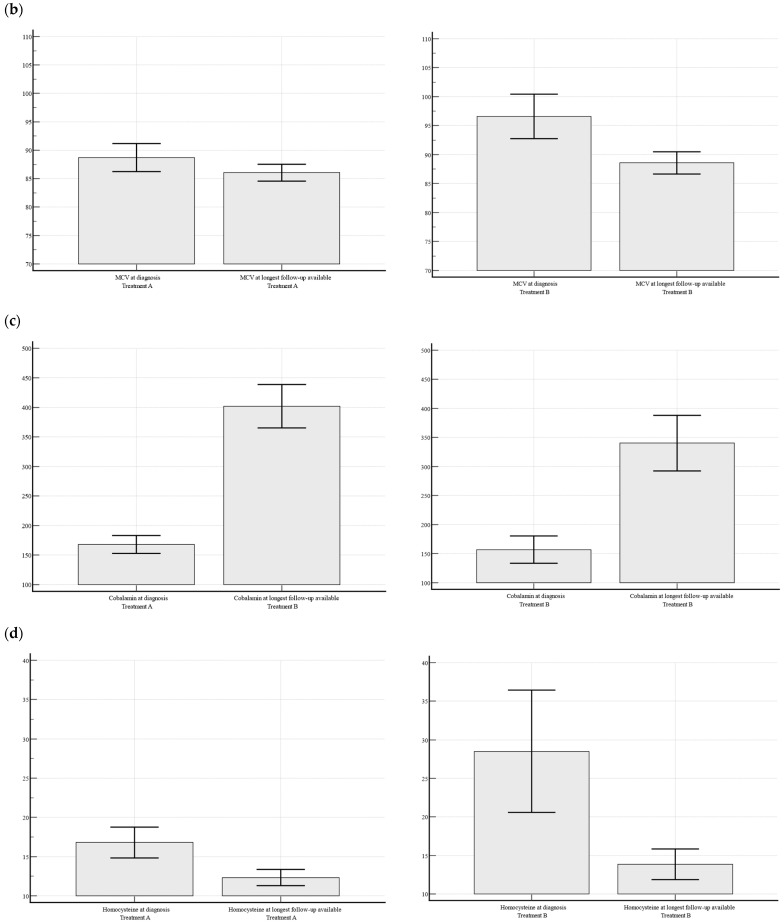
Variations in hematological (hemoglobin, Hb, g/dL), and mean corpuscular volume, (MCV, fl), and serological (serum cobalamin, pg/mL, and homocysteine, μmol/L) parameters from the start of treatment to the longest available follow-up of the 1st treatment (TxA) and the 2nd treatment schedule (TxB). TxA consisted of 5000 µg IM cyanocobalamin every five days for three times, followed by 5000 µg IM every three months (20,000 µg yearly). TxB consisted of 5000 µg IM cyanocobalamin every five days for three times followed by 5000 µg IM cyanocobalamin every two months (30,000 µg yearly). (**a**) Variation in mean ± SEM hemoglobin (Hb) levels (g/dL) at baseline and follow-up in TxA (left graph, *p* < 0.001) and B (right graph, *p* = 0.003). (**b**) Variation in mean ± SEM mean corpuscular volume (MCV, fl) at baseline and follow-up in TxA (left graph, *p* = 0.069) and B (right graph, *p* = 0.0003). (**c**) Variation in mean ± SEM serum cobalamin levels (pg/mL) at baseline and follow-up in TxA (left graph, *p* < 0.0001) and B (right graph, *p* < 0.0001). (**d**) Variation in mean ± SEM serum homocysteine levels (μmol/L) at baseline and follow-up in TxA (left graph, *p* = 0.0001) and B (right graph, *p* = 0.0002). All variations were compared by Student’s *t* test for paired variables.

Figure 1 illustrates the variations in hematological and biochemical parameters from the start of each treatment schedule to the longest available follow-up.

Table 1 presents a comparison of the main clinical, biochemical, and histological features in univariate analysis. CAG patients with vitamin B_12_ deficiency who needed the TxB schedule were significantly more frequently males (49.2% vs. 32.9%, *p* = 0.023), had more frequently macrocytosis at diagnosis (40.6% versus 19.9%, *p* = 0.002), severe corpus intestinal metaplasia (20.9% versus 2.8%, *p* < 0.0001), and had lower serum cobalamin levels at diagnosis (mean ± SEM, 137 ± 10 versus 165 ± 8 pg/mL, *p* = 0.041). At multivariate analysis (logistic regression) the presence of severe corpus intestinal metaplasia (OR 11.0, 95% CI 2.8 to 43.7), the presence of macrocytosis at CAG diagnosis (OR 2.7, 95% CI 1.2 to 6.3), and male sex (OR 2.4, 95% CI 1.1 to 5.2) were significantly associated with the need of TxB schedule to achieve normalization of serum cobalamin levels, while other variables such as age at CAG diagnosis, previous cobalamin supplementation treatment, serum cobalamin levels less than 100 pg/mL, or concomitant iron deficiency were not associated. Beyond the association of male sex with TxB, no further gender differences were observed in this study.

## 4. Discussion

To the best of our knowledge, this is the first study to assess the efficacy of high-dose IM cyanocobalamin treatment in patients with CAG along with concomitant vitamin B_12_ deficiency.

It is well known that CAG, more commonly in its corpus-restricted form (autoimmune atrophic gastritis), but also in its extended form to the antral mucosa, may be associated with vitamin B_12_ (cobalamin) deficiency. This is a physiopathological consequence of impaired gastric acid secretion and intrinsic factor deficiency resulting from corpus atrophy [6,9]. Untreated vitamin B_12_ deficiency can lead to megaloblastic anaemia (known as pernicious anemia), severe neurological consequences, and high homocysteine levels with increased risk of acute cardio-cerebral and vascular events [7,8,9]. Therefore, timely and efficacious vitamin B_12_ supplementation is a clinically relevant point. In vitamin B_12_ deficiency related to CAG, the IM route of administration is recommended to overcome intrinsic factor deficiency and intestinal malabsorption [12,13,14]. The optimal dosage of IM cyanocobalamin in this specific patient setting has not been investigated, and empirical treatment schedules are generally used. A frequently used schedule consists of 1000 μg cyanocobalamin or hydroxocobalamin IM every other day for one to two weeks, continuing then with weekly IM injections for one month and finally with once-a-month injections lifelong [14]. In our clinical practice, accounting for a long-lasting experience in treating patients with cobalamin deficiency, the schedule consists of administering a higher dosage of cyanocobalamin (5000 μg), but less frequent injections, starting with an initial load of three injections every five days and then continuing with one injection every three months for a lifetime. This schedule was adopted to simplify supplementation by reducing the number of injections and increasing feasibility and comfort. Another clinical-pharmacological reason for adopting this high-dosage schedule was that only about 10% to 15% of the injected dose (for example, 100–150 μg of the injected 1000 μg) is retained, and increasing the dosage should lead to retaining a higher amount of cobalamin, contributing to a quicker repletion of stores [13,14].

One of the main findings of the current study was that, by adopting this treatment schedule, nearly 70% of patients with CAG and vitamin B_12_ deficiency could permanently restore their serum vitamin B_12_ levels. This finding was robustly supported by the significant increases in hemoglobin and serum cobalamin levels, as well as the significant decreases in serum homocysteine levels, at long-term follow-up compared to the start of treatment. Even if the IM injection of cyanocobalamin is painful and requires assistance from a caregiver or nurse, this treatment schedule, consisting of only four IM injections per year, can be easily organized, being feasible and comfortable, and leading to normal serum cobalamin levels for an extended period of time.

Conversely, another significant result of the current study was that a subgroup of patients with CAG did not restore their serum vitamin B_12_ levels with the abovementioned schedule, but needed a higher amount of cyanocobalamin to achieve this endpoint as they normalized their serum vitamin B_12_ levels with 5000 µg IM cyanocobalamin injections every two months (30,000 µg yearly), after an initial load of 5000 µg IM cyanocobalamin every five days for three times. Also for this schedule, the efficacy was supported by a significant increase in hemoglobin and serum cobalamin levels, as well as a significant decrease in MCV and serum homocysteine at long-term follow-up compared to the start of treatment. Interestingly, the increased need for cyanocobalamin to restore normal serum vitamin B_12_ levels was associated with the male sex (OR 2.4), the presence of macrocytosis at diagnosis (OR 2.7) and the presence of severe corpus intestinal metaplasia (OR 11.0).

However, patients were switched to the 2nd treatment schedule based on persistent vitamin B_12_ deficiency; however, the follow-up interval at which this decision was made varied, ranging from 6 to 36 months. This variability raises the possibility that some patients might have shown a delayed response rather than true treatment failure. In clinical practice, in presence of biochemical evidence of vitamin B_12_ deficiency, action is required as untreated vitamin B_12_ deficiency may lead to underhand consequences. For this reason, in the current study, treatment was switched to the higher dosage as soon as evidence of persistent vitamin B_12_ deficiency was observed, without watching and waiting for a later treatment response, which, however, cannot be excluded.

While autoimmune atrophic gastritis occurs more commonly in females, pernicious anemia is associated with a higher prevalence in males [18]. A previous study, exploring the gender differences in homocysteine concentrations on 9237 men and 4353 women showed that average homocysteine concentrations were significantly higher in men than in women (*p* < 0.001) and that homocysteine levels above 15 μmol/L were found to be significantly higher in men than in women (15.5% vs. 3.9%, respectively, *p* < 0.001), along with low vitamin B_12_ (<200 pmol/L) levels which were significantly more common in men than in women. Compared to females, males had a significantly higher OR, 95% CI of having homocysteine concentrations above 15 μmol/L (4.5, 3.8–5.3) and low serum vitamin B_12_ levels (3.4, 2.9–4.1) [19]. Therefore, intrinsic, sex-related or behaviour-environment-related gender differences might contribute to an accelerated cobalamin metabolism leading to a higher cobalamin need in males. However, we cannot exclude that sex-related differences might be influenced by factors that were not fully captured in the dataset.

The presence of macrocytosis can be interpreted as an indirect sign of the severity of vitamin B_12_ deficiency. One of the significant complications of vitamin B_12_ deficiency is megaloblastic anaemia, resulting from the inhibition of DNA synthesis. Albeit all formed blood elements are influenced by the ineffective megaloblastic hematopoiesis, erythrocytes show the most evident changes regarding size and shape, with large oval macrocytes and anisopoikilocytosis; mild macrocytosis may be the first sign of a megaloblastic process, but a gradual shift in MCV occurs as merging with older normocytic erythrocytes occurs due to the longevity of these cells; finally macrocytic anemia occurs (MCV > 100 fl) [14]. The link between macrocytosis and the severity of cobalamin deficiency might explain the increased cobalamin requirements in CAG patients with macrocytosis at diagnosis. In this context, it is worth noting that CAG may sometimes be associated with dimorphic anemia as a result of the concomitant presence of iron deficiency (causing microcytosis) and cobalamin deficiency (causing macrocytosis), resulting in normocytic anemia [9,20]. It has also been reported that in CAG, cobalamin deficiency occurs at a later stage compared to iron deficiency generally present at an earlier point of the natural history [5,21]; therefore, the “pure” cobalamin deficiency may be a result of a more severe and advanced corpus atrophy and intrinsic factor deficiency requiring higher amounts of cobalamin to replenish physiological stores. Indeed, the presence of severe corpus intestinal metaplasia also served as a predictor of an increased requirement for cobalamin to normalize serum vitamin B_12_ levels. Corpus intestinal metaplasia represents a histopathological marker of advanced, severe, and longstanding disease, testifying to the substitution of the native gastric oxyntic glands with metaplastic cells resembling those of the intestine, leading to achlorhydria and absence intrinsic factor secretion, thus explaining the increased need for cobalamin supplementation. In this context and from a broader perspective, macrocytosis and severe intestinal metaplasia may be viewed as indicators of patients at higher risk for more severe disease, rather than merely predictors of a higher cobalamin dosage requirement.

We are aware of some limitations of our study. Unfortunately, symptoms related to vitamin B_12_ deficiency were not systematically collected. In this study, the normalization of serum vitamin B_12_ was the primary endpoint. Although this is a reasonable and clinically relevant measure, vitamin B_12_ levels alone may not fully reflect functional repletion. The improvements in hemoglobin, MCV, and homocysteine are reassuring, but neurological symptoms and other clinical manifestations of deficiency may sometimes be clues to functional deficiency even in the presence of normal serum cobalamin levels. This study provides supportive evidence for the recovery of hematological and biochemical markers; however, it does not offer definitive proof for the improvement of clinical indicators. At long-term follow-up, adverse events possibly associated with IM cyanocobalamin supplementation were not observed. All patients who adhered to long-term treatment were invited to annual clinical visits, and none of them interrupted their injections until the longest available follow-up. However, among dropouts, one patient stopped cyanocobalamin injections after the first administration due to side effects at the site of injection, continuing then with oral supplementation. This is not surprising, as the toxicity of cobalamin has been reported as minimal, and allergic reactions appear to be uncommon, occurring less frequently with cyanocobalamin compared to other molecular forms [22]. In this study, we did not administer other dosages, beyond the dosage used in our clinical practice, or other molecular forms of cobalamin for comparison. The reason for this is the real-life setting in which the most commonly available drug, namely cyanocobalamin, was prescribed. However, previous work did not show advantages of methylcobalamin or other coenzyme forms over cyanocobalamin [23], but these data derive from different settings not directly comparable to our study. Of the initial 710 CAG patients, 497 were excluded; however, 210 (30%) were excluded due to a lack of vitamin B_12_ deficiency. Nevertheless, a potential selection bias cannot be ruled out. Interestingly, the use of metformin or MTHFR mutations did not differ between patients in the two treatment schedules; this may be because their overall prevalence was only about 10% in both groups, thus representing a relatively small number of patients.

## 5. Conclusions

In this real-world setting, the current study demonstrates that in CAG patients with vitamin B_12_ deficiency, high-dosage intramuscular cyanocobalamin treatment with a reduced number of injections can restore serum cobalamin levels at long-term follow-up. Nearly 70% of patients restored serum vitamin B_12_ levels with 20,000 µg/yr IM cyanocobalamin (four injections of 5000 µg yearly), while the remaining 30% required 30,000 µg/yr (six injections of 5000 µg yearly). Male sex, advanced gastric damage and macrocytosis at the time of CAG diagnosis were predictors for higher need of cyanocobalamin. CAG patients with these characteristics should be carefully monitored to avoid suboptimal supplementation and potentially dangerous consequences of vitamin B_12_ deficiency.

## Figures and Tables

**Table 1:** Comparison of main clinical and histological features of patients with corpus atrophic gastritis and associated cobalamin (vitamin B_12_) deficiency treated with the 1st treatment (TxA) and the 2nd treatment schedule (TxB). The first treatment schedule (TxA) consisted of 5000 µg IM cyanocobalamin every five days for three times, followed by 5000 µg IM every three months (20,000 µg yearly). The 2nd treatment schedule (TxB) consisted of 5000 µg IM cyanocobalamin every five days for three times, followed by 5000 µg IM cyanocobalamin every two months (30,000 µg yearly).

	All Patients n = 213	Patients Treated with TxA n = 146 (68.5%)	Patients Treated with TxB n = 67 (31.5%)	*p*
**Clinical-biochemical features**				
Females	62.9%	67.1%	50.8%	0.022
Age, years, median (range)	63 (20–86)	62 (20–86)	65 (33–85)	0.748
Presence of anemia	48.3%	50.0%	44.6%	0.273
Severity of anemia				
-severe	7.8%	7.8%	7.8%	0.556
-moderate	8.8%	10.6%	4.7%	
-mild	31.5%	31.7%	31.3%	
-absent	51.9%	50.0%	56.3%	
Presence of macrocytosis	26.3%	19.9%	40.6%	0.002
Serum cobalamin levels, pg/mL, mean ± SEM	164 ± 6	165 ± 8	137 ± 10	0.041
Serum cobalamin levels				
<200 pg/mL	41.7%	43.9%	37.1%	0.250
<100 pg/mL	28.1%	23.9%	37.1%	
Cobalamin supplementation before standard treatment	73.6%	72.4%	76.1%	0.570
High serum homocysteine levels	60.5%	58.8%	64.1%	0.578
Concomitant iron deficiency	54.8%	57.6%	48.0%	0.250
Body mass index, kg/m^2^, mean ± SEM	25.7 ± 0.3	25.2 ± 0.4	26.3 ± 0.8	0.171
Smoking				
-Active	21.5%	21.2%	22.2%	0.377
-Past	20.0%	17.5%	25.4%	
-Never	58.5%	61.3%	52.4%	
Vegetarian diet	0.5%	0.7%	0.0%	0.495
Autoimmune diseases				
-absent	58.1%	56.6%	61.5%	0.499
-autoimmune thyroid disease (AITD)	28.1%	29.7%	24.6%	
-other autoimmune (AI) diseases	6.7%	5.5%	9.2%	
-AITD and other AI diseases	7.1%	8.3%	4.6%	
Diabetes				
-type I	2.8%	2.7%	3.0%	0.781
-type II	13.1%	13.7%	11.9%	
-not specified	0.9%	1.4%	0.0%	
Myelodysplasia	1.4%	0.7%	3.0%	0.187
Mthfr mutation	9.0%	8.3%	10.5%	0.618
Metformin treatment	11.4%	11.7%	10.6%	0.813
Aspirin treatment	14.6%	15.2%	13.4%	0.739
Use of >3 chronic drug treatments	14.8%	16.8%	10.6%	0.244
Previous abdominal surgery	6.7%	6.3%	7.6%	0.721
**Gastric histopathological features**				
Corpus-restricted atrophy	73.4%	73.1%	74.2%	0.862
Corpus atrophy severity score				
-Mild	9.5%	10.4%	7.6%	0.197
-Moderate	16.6%	19.3%	10.6%	
-Severe	73.9%	70.4%	81.8%	
Corpus intestinal metaplasia severity score				
-Absent	13.2%	15.2%	9.0%	<0.0001
-Mild	40.1%	45.5%	28.4%	
-Moderate	38.2%	36.6%	41.8%	
-Severe	8.5%	2.8%	20.9%	
*Helicobacter pylori* positivity at histology	31.9%	29.5%	37.3%	0.254
Gastric neoplastic lesions (diagnosed at baseline or follow-up)	10.8%	11.0%	10.5%	
-Type 1 gastric neuroendocrine tumour	6.1%	6.9%	4.6%	0.911
-Gastric cancer	4.7%	4.1%	5.9%	0.966

Data are expressed as percentages when not otherwise indicated. Statistical comparisons were performed by the Chi-square test for qualitative variables and by Student’s *t*-test for continuous variables.

## Data Availability

The dataset supporting the conclusions of this article will be made available by the authors on request according to the local policy.
